# Hyaluronic Acid Molecular Weight in the Minimally Invasive Management of Temporomandibular Disorders: A Scoping Review

**DOI:** 10.3390/jcm15197597

**Published:** 2026-09-30

**Authors:** Maja Kosińska, Maciej Chęciński, Wojciech Macek, Klaudia Kwiatkowska, Karolina Grzybowska-Kowalczyk, Tomasz Horodniczy, Amelia Hoppe, Julia Kasprzycka, Oliwia Jagiełło, Zuzanna Baniak, Kamila Chęcińska, Maciej Sikora

**Affiliations:** 1 Department of Oral Surgery, Preventive Medicine Center, Komorowskiego 12, 30-106 Cracow, Poland; majaakosinska@gmail.com (M.K.); ld.wojciech.macek@wp.pl (W.M.); amelia.a.hoppe@gmail.com (A.H.); 2 National Medical Institute of the Ministry of the Interior and Administration, Wołoska 137, 02-507 Warsaw, Poland; lek.dent.k.kwiatkowska@wp.pl (K.K.); maciej.sikora@pimmswia.gov.pl (M.S.); 3 Department of Maxillofacial Surgery, Hospital of the Ministry of the Interior and Administration, Wojska Polskiego 51, 25-375 Kielce, Poland; 4 Uśmiech Family Dental Clinic, Nastrojowa 26, 91-496 Łódź, Poland; karolina.orto.gk@gmail.com; 5 Ortho.pl Dental Office, Buforowa 34, 52-131 Wrocław, Poland; tomasz.horodniczy2@gmail.com; 6 Miodowa Clinic, Głowaccy Medical and Dental Practice Professional Partnership, Kiekrz, Miodowa 2, 62-090 Rokietnica, Poland; lekdentkasprzycka@gmail.com; 7 Gabinety Lekarskie Dent-im M.B. Pawelczyk Spółka Jawna, Różana 13/1, 61-577 Poznań, Poland; oliwiajagiellodent@gmail.com; 8 Stomatologia Centrum, Moniuszki 3/7, 32-020 Wieliczka, Poland; baniakzuzanna@gmail.com; 9 Department of Biochemistry and Medical Chemistry, Pomeranian Medical University, Powstańców Wielkopolskich 72, 70-111 Szczecin, Poland

**Keywords:** temporomandibular joint disorders, hyaluronic acid, molecular weight, intra-articular injections

## Abstract

**Background/Objectives**: This scoping review mapped how hyaluronic acid (HA) molecular weight has been defined and investigated in direct clinical comparisons for temporomandibular disorders (TMDs), including formulations, populations, procedures, outcomes, and methodological limitations. **Methods**: Final searches and selection were completed on 9 September 2026 using PubMed, BASE, CENTRAL, and Europe PMC. Prospective human studies comparing different intra-articular HA preparations under the same procedural protocol were eligible, including matched comparisons within multi-arm studies. Two reviewers independently screened and charted the evidence. Findings were synthesized descriptively without meta-analysis or formal risk-of-bias assessment. The protocol was registered in OSF (osf.io/sc8wv). **Results**: Five reports were included: three quasi-randomized trials and two prospective studies with sequential or unreported allocation between HA formulations. Four reported efficacy comparisons; one contributed safety data only after swelling and marked pain in 2 of 5 recipients prompted discontinuation of a high-molecular-weight arm. Molecular-weight categories overlapped across reports, formulation characteristics were incompletely reported, and protocols varied from one to five injections with differing use of arthrocentesis. Comparative follow-up ranged from 3 to 12 months. No eligible efficacy comparison detected a statistically significant difference, but small samples, allocation limitations, and incomplete subgroup reporting restricted interpretation. **Conclusions**: The evidence map identifies inconsistent formulation classification, procedural heterogeneity, and deficiencies in outcome and safety reporting as barriers to interpretation. It does not establish equivalence, superiority, or comparative safety of molecular-weight categories and provides specific priorities for future studies.

## 1. Introduction

### 1.1. Rationale

Intra-articular hyaluronic acid (HA) is used in minimally invasive management of joint-related temporomandibular disorders (TMDs), often together with arthrocentesis. Existing reviews describe a range of HA injection approaches, with variation in patient selection, accompanying procedures, and clinical outcomes [1,2,3,4]. This diversity makes the properties of the injected preparation relevant to the interpretation of treatment studies. Evidence about HA as an intervention does not, by itself, establish how preparations with different molecular weights compare.

Goiato et al. reported benefits for pain and function following intra-articular HA but emphasized that the appropriate injection protocol, number of sessions, and molecular weight remained to be established [4]. Ferreira et al. reviewed 21 articles and found that heterogeneity in HA formulations and administration protocols, together with methodological inconsistencies, prevented firm conclusions about efficacy in articular TMDs [3]. Derwich et al. subsequently concluded that adding HA to arthrocentesis did not improve clinical outcomes compared with arthrocentesis alone [2]. Together, these findings provide a rationale for evaluating formulation characteristics alongside procedural factors.

HA contributes to the viscoelastic properties of synovial fluid and participates in cell signaling and tissue homeostasis. Its biological effects depend on polymer size and the surrounding tissue environment [5,6,7]. Experimental work on fragmented hyaluronan, including Toll-like receptor signaling, provides a rationale for investigating molecular-weight-dependent activity [8]. However, endogenous fragments generated during inflammation and commercial preparations described as low-molecular-weight HA are not interchangeable biological exposures. Mechanistic observations cannot establish which injected formulation produces better clinical outcomes in the TMJ.

A useful clinical evidence map must therefore describe more than the labels low, medium, and high molecular weight. Absolute molecular-weight values or ranges, product identity, concentration, and cross-linking are needed to characterize the formulations being compared. The interpretation of a comparison also depends on diagnosis, baseline impairment, injection frequency and volume, arthrocentesis, accompanying conservative treatment, outcome definitions, and follow-up. Mapping these features can distinguish gaps in the availability of studies from gaps in their design and reporting.

This review focuses on direct comparisons of different HA preparations delivered under the same procedural protocol within each study. Such comparisons reduce some procedural differences between groups, while allowing the review to examine how formulation characteristics and study methods have been reported. The purpose is to characterize this comparative evidence and its interpretive limits, without assuming that the available studies can isolate the effect of molecular weight or support a ranking of HA products.

### 1.2. Objectives

The primary question was: how have HA formulations differing in molecular weight been defined, compared, and evaluated in prospective studies of minimally invasive treatment for TMJ disorders? The population was people with clinically diagnosed TMJ disorders; the concept was comparison of distinct intra-articular HA preparations; and the context was a shared procedural protocol within each comparison. The objectives were to map molecular-weight definitions and formulation reporting, diagnostic and population characteristics, injection and arthrocentesis protocols, outcome measures, follow-up, allocation and blinding methods, and safety reporting, and to identify the resulting priorities for future comparative research.

## 2. Materials and Methods

### 2.1. Protocol and Registration

The review protocol was prospectively registered in the Open Science Framework (OSF) under registration number: osf.io/sc8wv. The review was conducted and reported in accordance with the PRISMA Extension for Scoping Reviews (PRISMA-ScR) (File S1) [9].

A scoping review design was selected to characterize the extent, terminology, methods, and reporting of the available comparative evidence. The unit of inclusion was a published prospective study report containing an eligible HA comparison; the unit of clinical synthesis was the protocol-matched comparison within that report. The mapping objectives included nonrandomized evidence and formulation-reporting gaps, rather than restricting the review to studies suitable for an effectiveness meta-analysis.

The final searches and selection were completed on 9 September 2026 after repetition of the preliminary searches and screening. Relative to the registered protocol, CENTRAL was added and eligibility was broadened from randomized trials to prospective comparative studies, including quasi-randomized and nonrandomized designs. Eligibility also explicitly permitted protocol-matched HA comparisons within multi-arm reports. These amendments served the evidence-mapping objective by capturing relevant formulation comparisons while retaining the requirement for the same procedural protocol within each comparison.

### 2.2. Eligibility Criteria

Study design and publication type: Prospective comparative human studies were eligible, including randomized, quasi-randomized, and nonrandomized designs. Studies had to compare distinct HA preparations under the same procedural protocol, defined by the injection technique, number and interval of administrations, volume, site, concomitant procedures, and follow-up schedule. A multi-arm report was eligible if it contained at least one qualifying HA comparison, regardless of the interventions in its other arms. Retrospective and noncomparative studies, case reports, and sources other than published journal articles were excluded.

Population: Studies enrolling people of any age with clinically diagnosed temporomandibular joint (TMJ) disorders were eligible, provided participants were allocated to different HA products used within the same protocol. Studies of mixed populations were excluded if TMJ-specific data were not reported separately. Non-human studies were excluded.

Intervention: Eligible interventions were intra-articular HA injections into the TMJ comparing distinct preparations that could differ in molecular weight, concentration, cross-linking, or brand. These attributes were charted separately; a difference between products was not assumed to represent a difference in molecular weight alone.

Comparison: Only head-to-head HA comparisons with the same procedural protocol were charted. Comparisons against placebo, saline, other agents, or other procedures were excluded, as were HA comparisons with different injection volumes, numbers of administrations, techniques, or concomitant procedures. Such ineligible arms did not exclude an otherwise eligible comparison within the same report.

Outcomes: At least one clinical or safety outcome was required. Clinical outcomes included pain measured using a visual analogue scale (VAS) or numerical rating scale (NRS), mandibular function, maximum mouth opening, or patient-reported global improvement. Safety outcomes included adverse events and complications. Follow-up timing was charted for each eligible comparison.

Language: No language restrictions were applied.

Requiring a shared procedural protocol within each comparison reduced confounding by treatment technique and schedule. It did not ensure that molecular weight was the only formulation attribute differing between groups. Table 1 summarizes the final eligibility criteria.

Trials using alternation or a fixed repeating allocation sequence were classified as quasi-randomized, irrespective of the original authors’ designation. Prospective groups treated in successive calendar periods were classified as nonrandomized. Allocation and blinding were recorded as not reported when the source did not describe them, including allocation between the eligible HA subgroups of a larger multi-arm study.

### 2.3. Information Sources

Information was obtained from PubMed, BASE, the Cochrane Central Register of Controlled Trials (CENTRAL) within the Cochrane Library, and Europe PMC. The final search across these four information sources was completed on 9 September 2026. Searches were performed by T.H. and M.C.

### 2.4. Search

The search combined terms for the TMJ, HA, intra-articular procedures, comparative studies, and formulation characteristics. The final database-specific strategies are reproduced in Appendix A.

### 2.5. Selection of Sources of Evidence

Records from all four databases were pooled in Rayyan (web version; Rayyan Systems, Inc., Cambridge, MA, USA; accessed in September 2026), and duplicates were removed (T.H.). The complete deduplicated record set was screened in two stages using Rayyan. Before formal screening, the reviewers completed a calibration exercise. Titles and abstracts were independently reviewed in a blinded manner (J.K. and O.J.), followed by independent full-text assessment against the final eligibility criteria (J.K. and O.J.). Reports with insufficient information to determine eligibility from the title and abstract were retained for full-text assessment. Disagreements were resolved through discussion and consensus, and reasons for exclusion at the full-text stage were recorded. Final selection was completed on 9 September 2026. The study selection process was documented in the flow diagram [10].

### 2.6. Data Charting Process

Data from the included studies were charted using a standardized data-charting form developed by the research team. Two reviewers (J.K. and O.J.) independently charted the data using tables prepared in Google Sheets (web version; Google LLC, Mountain View, CA, USA; accessed in September 2026). Any discrepancies were resolved through discussion and consensus.

### 2.7. Data Items

Charted items comprised study design and setting; participant and eligible subgroup sizes; diagnostic criteria, baseline characteristics, and TMD subtype or severity; HA product and manufacturer; molecular-weight category and absolute value or range in kDa; concentration and cross-linking; injection site, volume, number, and interval; arthrocentesis technique and lavage volume; concomitant treatment; allocation and blinding; outcome instruments and definitions; follow-up; adverse events; and reported clinical findings. Patient counts, joint counts, and outcome-analysis denominators were distinguished. Molecular weights and categories were retained as reported, with unreported details labeled accordingly. Values read from figures and subgroup sizes inferred from reported totals were identified explicitly. Product information was checked separately when an apparent inconsistency in molecular-weight reporting was identified; this external check did not replace the study-reported exposure data.

### 2.8. Synthesis of Results

Findings were synthesized descriptively across formulation definitions, populations, procedures, outcomes, follow-up, and study methods. Efficacy and safety findings were considered separately, and only eligible comparisons were included from multi-arm studies. No quantitative pooling or ranking of formulations was undertaken. Formal risk-of-bias and certainty-of-evidence assessments were not performed; critical appraisal is optional under PRISMA-ScR [9]. Reported allocation, blinding, analysis, and safety features were mapped to identify limits to interpretation, without assigning overall quality grades. Nonsignificant comparisons were not interpreted as demonstrating equivalence.

## 3. Results

### 3.1. Selection of Sources of Evidence

Searches of four information sources identified 695 records: 177 from PubMed, 360 from BASE, 87 from CENTRAL, and 71 from Europe PMC. After removal of 378 duplicates, 317 unique records underwent screening.

During screening, the reviewers agreed to retain four records and exclude 308 records, with disagreements concerning five records. Observed agreement was 98.4% (312/317), and Cohen’s kappa, calculated from the initial screening decisions across all 317 records, was 0.609. The predominance of exclusion decisions resulted in highly imbalanced marginal distributions, increasing expected chance agreement and reducing kappa despite the high observed agreement. All nine records retained by at least one reviewer proceeded to eligibility assessment: seven journal articles, one dissertation, and one trial registry entry.

Eligibility was assessed using the full-text reports and the registry record. Four sources were excluded: one because the intervention combined HA with chitosan, one because the comparison involved different doses of the same HA preparation, and two because of ineligible publication types (one dissertation and one trial registry entry).

Five journal articles were included in the scoping review. Four contributed to the mapping of comparative efficacy findings, while one contributed exclusively to the descriptive synthesis of safety because the relevant treatment arm had been discontinued and comparative efficacy data were unavailable. Only eligible comparisons between HA preparations were included from multi-arm studies.

**Figure 1:**
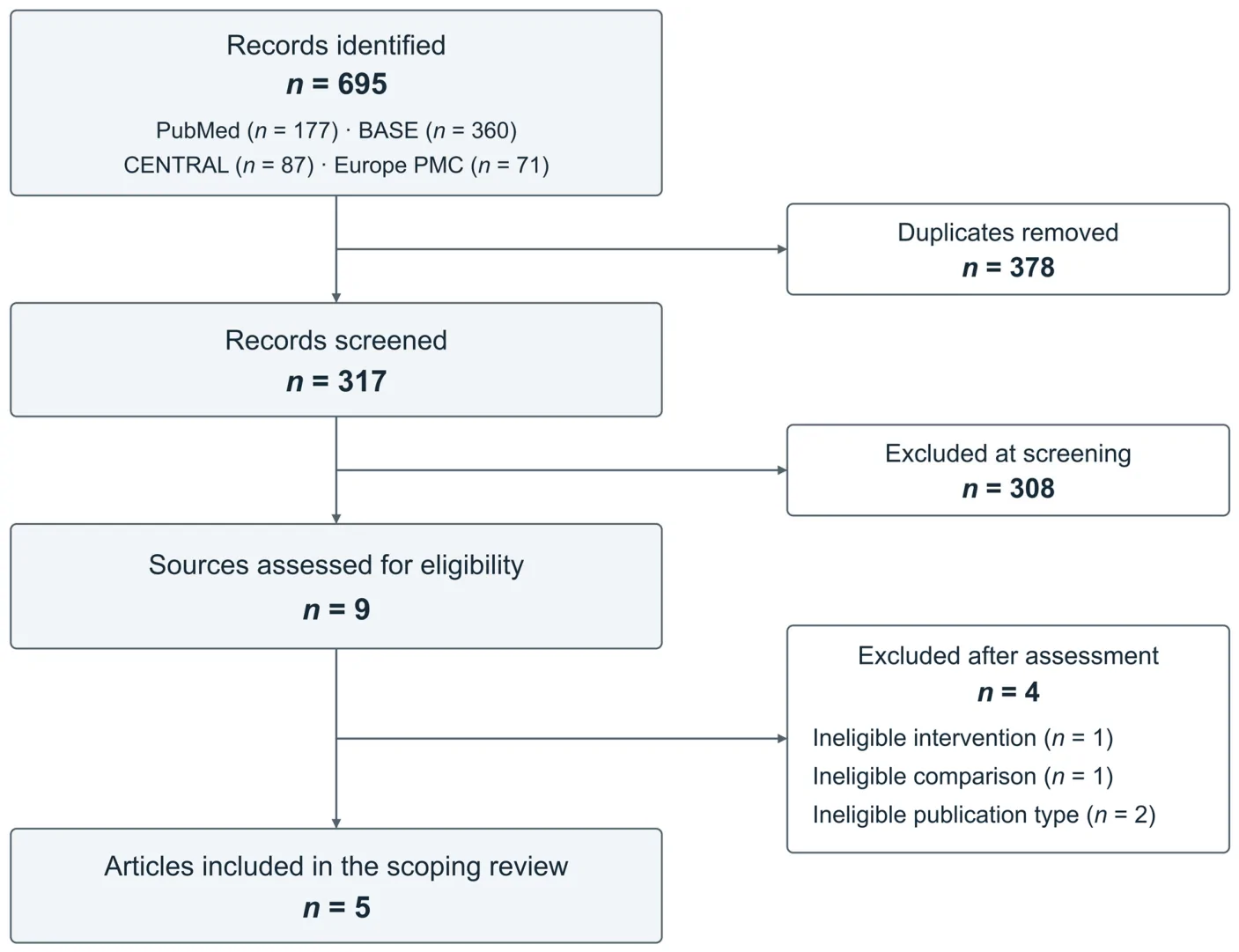
Flow diagram of study selection.

The study selection process is illustrated in Figure 1.

### 3.2. Characteristics of Sources of Evidence

Five prospective comparative reports published between 2012 and 2023 met the eligibility criteria. Three were quasi-randomized trials from the same Italian research group [11,12,13]; two were prospective comparative studies from a Hungarian center [14,15]. Four reports provided eligible efficacy comparisons: one low- versus medium-, one medium- versus high-, and two low- versus high-molecular-weight HA comparisons. The remaining low- versus high-molecular-weight comparison contributed safety data only.

Guarda-Nardini et al. (2012) compared medium-molecular-weight HA (Sinovial, 1200 kDa) with low-molecular-weight HA (Hyalgan, 600 kDa) in 40 patients with TMJ osteoarthritis [11]. Although the authors described the study as an “exploratory randomized clinical trial,” it was categorized as quasi-randomized in this review because allocation alternated between groups, resulting in a predictable sequence. Both groups underwent five weekly single-needle arthrocenteses using approximately 10 mL of saline, followed by 1 mL of HA. Thirty-five participants completed the 3-month follow-up. Participants and the outcome assessor were blinded, but the operators were not. Osteoarthritis was diagnosed using RDC/TMD axis I group IIIb, with joint pain lasting more than 6 months. The completed groups comprised 17 Sinovial and 18 Hyalgan recipients; their mean ages were 47.7 and 52.9 years, respectively.

Manfredini et al. (2012) allocated 72 patients with TMJ osteoarthritis among six treatment protocols using a fixed sequence [12]. The eligible molecular-weight comparison comprised identical single-session two-needle arthrocentesis using at least 300 mL of saline followed by 1 mL of low-molecular-weight HA (Hyalgan) or high-molecular-weight HA (Synvisc). The high-molecular-weight arm was discontinued after five participants because two developed joint swelling and marked post-injection pain; efficacy outcomes for this arm were not reported. The trial therefore contributed comparative safety information only. Patients had RDC/TMD axis I group IIIb osteoarthritis and pain for more than 6 months. Eleven participants completed the eligible low-molecular-weight HA protocol; the initial size of this arm was not separately reported. The outcome assessor was blinded, and patient blinding was attempted; operators were not blinded to the procedure performed.

Guarda-Nardini et al. (2015) enrolled 30 patients with chronic TMJ degenerative disorders into three groups using a fixed allocation sequence [13]. The eligible pairwise comparison involved two groups of 10 participants receiving identical single-session single-needle lavage with approximately 10 mL of saline followed by 1 mL of either high-molecular-weight HA (Durolane SJ, 7000 kDa, Bioventus, Durham, NC, USA) or medium-molecular-weight HA (Sinovial, 1200 kDa, IBSA Institut Biochimique SA, Lugano, Switzerland). All participants completed the 6-month follow-up, and no adverse events were reported. Eligibility required age 45–65 years, unilateral pain for more than 6 months, RDC/TMD axis I group IIIb osteoarthritis, and MRI evidence of degeneration. Patients with high pain-related disability on RDC/TMD axis II were excluded. The outcome assessor was blinded; operator blinding was not reported.

Vingender et al. (2018) [14] prospectively studied 24 patients with 37 affected joints, including 17 patients in the eligible HA comparison: 6 receiving low-molecular-weight HA (600–1000 kDa) and 11 receiving high-molecular-weight HA (2400–3600 kDa). HA products were allocated in successive calendar periods, from October 2013 to March 2014 and from April to September 2014, respectively. The overall cohort of 24 patients, including those receiving corticosteroids, had a reported mean age of 46 years and comprised 22 women and 2 men. The 17 patients receiving HA had a reported mean age of 47 years, but their sex distribution was not reported separately. Mean age and sex distribution were not reported separately for the groups receiving low- and high-molecular-weight HA. Blinding was not reported. Patients had persistent symptoms after conservative treatment, met RDC/TMD criteria, and had MRI-confirmed internal derangement; severity was assessed using the Wilkes classification. Both HA groups received three weekly 1-mL injections into the superior joint compartment. The procedure was described by reference to a lavage-based injection technique, but the lavage volume and needle configuration were not specified. Outcomes were assessed at 1 week and 6 months; an additional 12-month assessment in five patients did not provide separate HA-group results.

Vingender et al. (2023) [15] prospectively studied 68 patients with 109 joints between 2015 and 2020; the mean age was 53 ± 16 years, and 59 patients were women. The HA group comprised 28 patients, derived from the severity counts in Table 1 of the source article; the stated equal division between HA formulations implies 14 patients per group. HA-subgroup joint counts and the numbers included in each outcome analysis were not reported. Patients had RDC/TMD-defined, MRI-confirmed internal derangement persisting after 2–5 months of conservative treatment; severity was graded with the Fonseca Anamnestic Index. Treatment with HA, platelet-rich plasma (PRP), or injectable platelet-rich fibrin (I-PRF) was assigned in successive phases, but allocation between the two HA formulations and blinding were not described. The eligible comparison used Hyalgan and Euflexxa, reported as 500–1500 and 6000–7000 kDa, respectively (the Euflexxa value differs from product information; see Section 3.4), with three weekly 1-mL superior-compartment injections and no preceding arthrocentesis. Follow-up was at 6 and 12 months. Both Hungarian reports described continuing splint therapy and physiotherapy after the injections. The principal characteristics and findings of the included studies are summarized in Table 2.

**Table 2:** Principal characteristics and findings of the included studies.

Study	Participants	HA Preparations and Reported Molecular Weights	Shared Procedural Protocol	Follow-Up	Principal Findings
Guarda-Nardini et al. (2012) [11]	40 enrolled; 35 completed follow-up: 17 MMW and 18 LMW recipients.	Sinovial (IBSA): MMW, 1200 kDa; Hyalgan (Fidia Farmaceutici S.p.A., Abano Terme, Italy): LMW, 600 kDa.	Five weekly single-needle arthrocenteses, each using approximately 10 mL saline, followed by 1 mL HA.	3 months.	No statistically significant between-formulation differences detected.
Manfredini et al. (2012) [12]	72 reported overall; eligible comparison: 11 LMW completers and 5 HMW recipients.	Hyalgan (Fidia): LMW; Synvisc (Genzyme, Cambridge, MA, USA): HMW. Absolute molecular weights not reported.	One two-needle arthrocentesis using at least 300 mL saline, followed by 1 mL HA.	3 months planned.	Safety data only: HMW arm discontinued after swelling and marked post-injection pain in 2/5 recipients; comparative efficacy unavailable.
Guarda-Nardini et al. (2015) [13]	30 overall; eligible comparison: 10 HMW and 10 MMW recipients, all completing follow-up.	Durolane SJ (Bioventus): HMW, 7000 kDa; Sinovial (IBSA): MMW, 1200 kDa.	One single-needle lavage using approximately 10 mL saline, followed by 1 mL HA.	1 week, 3 months, and 6 months.	No statistically significant between-formulation differences detected.
Vingender et al. (2018) [14]	24 patients/37 joints overall; eligible HA comparison: 17 patients, comprising 6 LMW and 11 HMW recipients.	LMW: 600–1000 kDa; HMW: 2400–3600 kDa. Product names and manufacturers not reported.	Three weekly 1-mL injections into the superior joint compartment; lavage details incompletely reported.	1 week and 6 months.	No statistically significant between-formulation differences reported; exact HA-pairwise *p* values and confidence intervals not provided.
Vingender et al. (2023) [15]	68 patients/109 joints overall; 28 HA recipients, with 14 per formulation inferred from the reported equal division.	Hyalgan (Fidia): LMW, 500–1500 kDa; Euflexxa (Ferring Pharmaceuticals, Saint-Prex, Switzerland): HMW, 6000–7000 kDa, as reported.	Three weekly 1-mL injections into the superior joint compartment, without preceding arthrocentesis.	6 and 12 months.	No statistically significant between-formulation differences reported for pain or mouth opening; exact HA-pairwise *p* values and confidence intervals not provided.

Abbreviations: HA, hyaluronic acid; HMW, high molecular weight; kDa, kilodaltons; LMW, low molecular weight; MMW, medium molecular weight. Molecular-weight values and category labels are reproduced as reported in the source articles. For Euflexxa, the reported range of 6000–7000 kDa differs from the manufacturer’s range of 2400–3600 kDa; see Section 3.4. Overall study populations are distinguished from eligible HA comparisons. Detailed safety findings and methodological characteristics are presented in Table 3 and Table 4, respectively.

**Table 3:** Safety findings and adverse-event reporting in the eligible HA comparisons.

Study	HA Formulations and Reported Molecular-Weight Categories	Reported Adverse Events	Patient Numbers and Safety Denominators	Arm Discontinued Because of Adverse Events
Guarda-Nardini et al. (2012) [11]	Hyalgan: LMW; Sinovial: MMW	Transient anesthesia involving the temporal and zygomatic facial nerve regions in 3 patients; distribution between formulations not reported.	20 patients treated per formulation; 18 Hyalgan and 17 Sinovial recipients completed follow-up. Formulation-specific event counts were unavailable.	No
Manfredini et al. (2012) [12]	Hyalgan: LMW; Synvisc: HMW	Synvisc: joint swelling and marked post-injection pain in 2/5 recipients. Hyalgan: no side effects reported.	Synvisc: 5 exposed patients. Hyalgan: 11 completers; initial exposed-group size not separately reported.	Yes; the Synvisc arm was discontinued after 5 recipients.
Guarda-Nardini et al. (2015) [13]	Sinovial: MMW; Durolane SJ: HMW	No side effects reported.	10 patients per eligible formulation group; all 20 completed follow-up.	No
Vingender et al. (2018) [14]	Brands not reported; LMW and HMW preparations	Adverse-event results for the eligible HA comparison were not reported.	6 LMW and 11 HMW recipients; safety-analysis denominators not reported.	Not reported
Vingender et al. (2023) [15]	Hyalgan: LMW; Euflexxa: HMW	Adverse-event results for the eligible HA comparison were not reported.	14 patients per formulation, inferred from the reported data; safety-analysis denominators not reported.	Not reported

Abbreviations: HA, hyaluronic acid; LMW, low molecular weight; MMW, medium molecular weight; HMW, high molecular weight. Categories are reproduced as reported in the source articles. Counts refer to patients, not treated joints. Treatment and completion counts do not necessarily represent verified safety-analysis denominators.

**Table 4:** Methodological characteristics and reporting completeness of the eligible HA comparisons.

Study	Allocation Method	Blinding	Definition of Eligible HA Subgroups	Outcome-Analysis Denominators
Guarda-Nardini et al. (2012) [11]	Alternating allocation between two groups; quasi-randomized.	Outcome assessor blinded; patients not informed of HA identity; operators not blinded to the procedure.	Sinovial (MMW) and Hyalgan (LMW) identified; 20 patients treated per formulation.	Reported: 17 MMW and 18 LMW recipients completed follow-up and were analyzed.
Manfredini et al. (2012) [12]	Fixed A–B–C–D–E–F–F–E–D–C–B–A sequence; quasi-randomized.	Outcome assessor blinded; patient blinding attempted; operators not blinded to the procedure.	Eligible arms identified as Hyalgan (LMW; arm C) and Synvisc (HMW; arm D).	Partial: 11 LMW completers and 5 HMW recipients before arm discontinuation. Initial LMW count not stated; HMW efficacy outcomes unavailable.
Guarda-Nardini et al. (2015) [13]	Fixed A–B–C–C–B–A sequence; quasi-randomized.	Outcome assessor blinded; patients received generic treatment information, but blinding to HA identity was not explicitly described; operator blinding not reported.	Eligible single-session arms identified as Durolane SJ (HMW; arm A) and Sinovial (MMW; arm B).	Reported: 10 patients per eligible arm; all completed follow-up and were analyzed.
Vingender et al. (2018) [14]	Successive calendar periods: October 2013–March 2014 for LMW and April–September 2014 for HMW; nonrandomized.	Not reported.	Groups defined by molecular-weight ranges; brands and manufacturers not reported.	Six LMW and 11 HMW patients treated; analysis denominators not explicitly reported for individual outcomes and follow-up times.
Vingender et al. (2023) [15]	Allocation between HA formulations not reported; sequential treatment phases described only for HA, PRP, and I-PRF.	Not reported.	Hyalgan (LMW) and Euflexxa (HMW) identified; an equal division was stated, implying 14 patients per formulation.	Not reported by HA formulation or outcome, including after exclusion of patients with mouth opening >35 mm from the mouth-opening analysis. HA-subgroup joint counts also unavailable.

Abbreviations: HA, hyaluronic acid; HMW, high molecular weight; I-PRF, injectable platelet-rich fibrin; LMW, low molecular weight; MMW, medium molecular weight; PRP, platelet-rich plasma. Molecular-weight categories are reproduced as reported in the original articles. Reporting adverse events or their absence does not establish systematic ascertainment. This descriptive map does not constitute a formal risk-of-bias or certainty-of-evidence assessment. Systematic adverse-event ascertainment was not described in any of the five included reports.

### 3.3. Results of Individual Sources of Evidence

In Guarda-Nardini et al. (2012), both groups improved from baseline in pain during chewing and chewing efficiency at 3 months [11]. Improvements in other outcomes differed in their within-group statistical significance, but these tests did not compare the two HA preparations.

Between-group analyses of changes over time showed no statistically significant differences in pain during chewing (F = 0.056, *p* = 0.815), pain at rest (F = 0.383, *p* = 0.541), chewing efficiency (F = 0.050, *p* = 0.825), functional limitation (F = 0.268, *p* = 0.609), or maximum mouth opening (F = 0.003, *p* = 0.954). No statistically significant between-group differences were found in perceived treatment effectiveness (*p* = 0.879) or treatment tolerability (*p* = 0.116).

No serious adverse events were reported. Three patients experienced transient anesthesia involving the temporal and zygomatic facial nerve regions after an intervention; these events were described as minor and temporary.

In Guarda-Nardini et al. (2015), the groups receiving high- and medium-molecular-weight HA under identical single-session protocols showed no statistically significant differences at 1 week, 3 months, or 6 months [13]. The global *p* value for the difference in treatment effect was 0.93, and the combined *p* values at the respective observation points were 0.60, 0.74, and 0.94. No participants withdrew and no adverse events were observed. In the groups receiving high- and medium-molecular-weight HA, the mean within-group changes from baseline to 6 months were −1.9 and −1.4 VAS points for pain during jaw function and +3.4 and +3.3 mm for mouth opening, respectively. Between-group comparisons were reported as permutation-test *p* values; no between-group effect estimates or confidence intervals were provided. The superior results of the third, five-session arm were outside the eligible formulation comparison because its treatment schedule differed.

In Manfredini et al. (2012), comparative efficacy could not be assessed because the high-molecular-weight HA protocol was discontinued after joint swelling and marked post-injection pain occurred in 2 of the first 5 participants [12]. No adverse events were reported in the group receiving low-molecular-weight HA or the other treatment groups. Thus, this trial provides a clinically relevant safety signal but no estimate of comparative efficacy.

In Vingender et al. (2018) [14], the authors reported no statistically significant differences between low- and high-molecular-weight HA for the assessed outcomes, without providing exact HA-pairwise *p* values or confidence intervals. Figure 3 of the source article showed mean mouth opening increasing from 31.8 to 38.4 mm with low-molecular-weight HA and from 26.67 to 35.33 mm with high-molecular-weight HA at 6 months. Figure 4 showed mean pain VAS scores of 7.83, 1.33, and 1.50 for low-molecular-weight HA and 7.55, 2.36, and 3.10 for high-molecular-weight HA at baseline, 1 week, and 6 months, respectively. The numerical HMW mouth-opening increase stated in the narrative (8.4 mm) differed from that calculated from Figure 3 (8.66 mm); the displayed means are therefore retained here without reconciliation. Improvements in the combined HA group and comparisons with corticosteroid were not treated as evidence of a molecular-weight effect.

In Vingender et al. (2023) [15], mean mouth opening at baseline, 6 months, and 12 months was 26.6, 36.4, and 37.4 mm in the group receiving low-molecular-weight HA and 23.8, 31.6, and 34.2 mm in the group receiving high-molecular-weight HA. Corresponding mean pain VAS scores were 7.8, 2.7, and 0.9 versus 5.4, 0.8, and 1.1. The authors reported no statistically significant differences between the HA formulations in either outcome, but exact HA-pairwise *p* values and confidence intervals were not provided. Patients with baseline mouth opening greater than 35 mm were excluded from the mouth-opening analysis, and bilateral joints were evaluated as independent observations without a reported adjustment for within-patient correlation. The HA subgroup means were reported without measures of dispersion. Neither Hungarian report provided a systematic adverse-event account with HA-group denominators; absence of such reporting was not interpreted as absence of events.

### 3.4. Synthesis of the Evidence Map

Molecular-weight definitions overlapped across reports. The 1200-kDa preparation labeled medium molecular weight in the Italian trials lay within the 500–1500-kDa range labeled low molecular weight in the 2023 report [11,13,15]. Reported high-molecular-weight values included 2400–3600 kDa, 6000–7000 kDa, and 7000 kDa; Manfredini et al. reported a category and brand without a numerical value [12,13,14,15]. Euflexxa was assigned 6000–7000 kDa in the 2023 article, whereas manufacturer information specifies 2400–3600 kDa [15,16]. The article’s value was retained as reported, with this unresolved discrepancy flagged rather than substituting the manufacturer’s value for the study exposure. Concentration and cross-linking were not fully characterized alongside molecular weight in the intervention descriptions.

The eligible comparisons used the same procedural schedule within each report but differed across reports: one, three, or five injections; small-volume single-needle lavage, large-volume two-needle arthrocentesis, incompletely specified lavage, or no arthrocentesis; and follow-up from 3 to 12 months. The Italian trials enrolled patients with chronic osteoarthritis, whereas the Hungarian studies included a broader spectrum of MRI-confirmed internal derangement after unsuccessful conservative therapy. Pain was assessed at rest, during chewing or jaw function, or as an unspecified overall VAS rating. Mouth opening was measured in millimeters, but the 2023 analysis excluded patients opening more than 35 mm. Other outcomes included chewing efficiency, functional limitation, perceived efficacy, tolerability, and Wilkes stage. HA-subgroup denominators, blinding, and adverse-event reporting were incompletely described in some reports.

None of the four reports providing eligible efficacy data detected a statistically significant difference between the compared HA preparations. Given the small eligible comparison groups and incomplete reporting of precision, the available evidence does not reliably exclude clinically meaningful between-formulation differences. The absence of statistical significance is therefore inconclusive for comparative efficacy and should not be interpreted as equivalence or as evidence that HA molecular weight is clinically irrelevant.

Follow-up reached 12 months in one report, but allocation between its HA subgroups, outcome-analysis denominators, and dispersion around subgroup means were not provided [15]. Across the map, three comparisons used quasi-random allocation, one used successive calendar periods, and one did not describe allocation between HA formulations [11,12,13,14,15]. Safety findings comprised one prematurely stopped high-molecular-weight arm, transient events in one trial, no reported events in another, and insufficient reporting in the two Hungarian studies. These findings describe the available evidence without establishing equivalence or comparative safety.

Table 3 summarizes the safety findings, available patient denominators, and treatment-arm discontinuation reported for the eligible HA comparisons.

Table 4 summarizes the allocation methods, blinding, subgroup definitions, and reporting completeness of the eligible HA comparisons.

## 4. Discussion

### 4.1. Principal Findings and Contribution of the Evidence Map

Five prospective comparative reports were identified in the final searches and selection: three quasi-randomized trials and two prospective studies with sequential or unreported allocation between HA formulations [11,12,13,14,15]. Four contained eligible efficacy comparisons, while one contributed safety findings after early discontinuation of its high-molecular-weight arm. The evidence map therefore covers low- versus medium-, medium- versus high-, and low- versus high-molecular-weight comparisons. The larger scope of the mapped evidence does not overcome its limitations for causal inference.

The map identifies several reasons why these comparisons cannot be treated as a coherent test of molecular weight. Category boundaries overlap, absolute values are sometimes absent or inconsistent with product information, and potentially relevant formulation attributes are incompletely reported. Procedures are matched within eligible comparisons but differ substantially between reports. Diagnostic subgroups, outcome definitions, follow-up, and analysis populations also vary. These findings specify where standardization and more complete reporting are needed, beyond documenting a small number of studies.

All four efficacy reports described nonsignificant differences between eligible HA groups. This does not establish equivalence, and differences between within-group *p* values do not establish a between-group effect. In studies combining HA with arthrocentesis, improvement from baseline also cannot separate the contribution of HA from lavage, natural symptom variation, or accompanying care. The eligibility restriction improves procedural comparability within each report but does not turn a product comparison into an isolated test of molecular weight.

### 4.2. Formulation and Clinical Interpretation

A category label should not be treated as a standardized exposure. For example, the overlap between 1200 kDa labeled medium molecular weight and 500–1500 kDa labeled low molecular weight shows why results cannot be organized into a simple low-to-high hierarchy [11,13,15]. Assigning unreported concentration or cross-linking from a brand name would add assumptions about the actual preparation used. Future studies should identify the formulation sufficiently precisely to distinguish molecular weight from other product characteristics.

The discrepancy concerning Euflexxa introduces uncertainty about the molecular-weight contrast evaluated by Vingender et al. [15]. The manufacturer’s range of 2400–3600 kDa would imply a smaller separation from Hyalgan (500–1500 kDa) than the reported Euflexxa range of 6000–7000 kDa, although the ranges of the two products would remain nonoverlapping [15,16]. The reported nonsignificant findings concern this particular product comparison and do not establish equivalent efficacy across molecular-weight categories. Alongside the overlapping category definitions, this discrepancy emphasizes the need to interpret comparisons using absolute molecular-weight values and sufficiently detailed formulation characteristics.

Mechanistic studies provide a rationale for examining molecular weight but cannot resolve these clinical uncertainties. Laboratory work describes size-dependent hyaluronan signaling [5,6,7,8]. Iturriaga et al. compared the histopathological effects of low- and high-molecular-weight HA on cartilage and articular discs in a rabbit model of TMJ osteoarthritis [17]. These observations concern biological mechanisms and experimental conditions; they cannot establish a preferred clinical preparation or explain the adverse reactions in the prematurely stopped Synvisc arm. That signal arose in a small sample exposed to one product under one protocol and cannot be attributed to molecular weight alone or generalized to all high-molecular-weight HA.

### 4.3. Priorities for Comparative Research

Broader evidence syntheses show that these problems are not confined to HA molecular weight. An overview of TMJ injections and lavage highlighted variation in agents, injection sites and schedules, and lavage techniques [18]. An overview restricted to standalone injections likewise found heterogeneity in diagnoses, protocols, comparators, outcomes, and follow-up; with only three eligible meta-analyses, it could not establish comparative superiority or long-term effects [19]. Recent syntheses produced intervention rankings, but these remain provisional: one included only seven trials in its 6-month meta-analysis and found very-low- to moderate-certainty evidence, while a network meta-analysis of 38 trials and 13 interventions called for further rigorous studies [20,21]. These limitations support standardized diagnostic criteria, intervention protocols, comparators, outcomes, safety reporting, and follow-up in future trials.

Future comparisons should begin with a clearly specified formulation contrast and a reproducible shared procedural protocol. Reporting molecular-weight ranges, concentration, cross-linking, manufacturer, and the actual product administered would make it possible to assess which characteristics differ between groups. Arthrocentesis technique, lavage volume, injection schedule, and concomitant therapy should be held constant or explicitly evaluated as separate intervention components.

Eligibility and outcomes also require greater consistency. Trials should define the joint disorder and baseline severity, prespecify the pain context being measured, and report mouth-opening criteria and patient-reported function at common follow-up times. Patient numbers, treated joints, and analysis denominators should be reported for every formulation and outcome. Bilateral observations require analysis that accounts for their correlation. Concealed random allocation, feasible blinding, sample sizes based on a prespecified clinically relevant contrast, and between-group estimates with confidence intervals are needed to make future findings interpretable.

Safety requires prospective ascertainment with explicit definitions, timing, severity, withdrawals, and exposed-group denominators. Incomplete reporting of adverse-event collection methods, absent formulation-specific safety results in two reports, and early discontinuation of one treatment arm leave comparative tolerability unresolved. Table 5 specifies proposed minimum reporting items and design priorities to support reproducible intervention descriptions and interpretable comparisons of efficacy and safety.

### 4.4. Strengths and Limitations

The review used explicit eligibility criteria, four information sources, independent screening and charting, and comparison-level extraction from multi-arm reports. Mapping reported values separately from inferred denominators and external product information helps expose uncertainties in the primary literature. These features support transparency, but they do not establish the validity of the underlying clinical comparisons.

The evidence was concentrated in three reports from one Italian research group and two from one Hungarian center, limiting the diversity of settings and populations. Predictable allocation in the Italian trials, calendar-based treatment in the 2018 study, and unreported allocation between HA formulations in the 2023 study permit selection bias and confounding [11,12,13,14,15]. Blinding was incomplete or insufficiently described. Small eligible groups, baseline differences, missing outcome-analysis denominators, and absent dispersion estimates in the Hungarian subgroup data limit precision and cross-study interpretation (Table 4). Treating bilateral joints as independent and excluding patients with mouth opening above 35 mm further complicate interpretation of the 2023 results. Eligible comparative follow-up extended to 12 months in only one report.

The review was restricted to published journal articles identified through PubMed, BASE, CENTRAL, and Europe PMC. Scopus and Web of Science were not searched, and additional eligible reports may therefore have been missed. The coverage of the evidence map is consequently limited by the information sources and publication types considered. Final eligibility encompassed nonrandomized studies, so the evidence should not be described collectively as randomized trials. Formal risk-of-bias and certainty assessments were not undertaken; the narrative description of methodological features is not a substitute for either. Unresolved reporting discrepancies and inferred subgroup sizes remain limitations of the charted evidence.

Requiring a shared procedural protocol within each comparison necessarily narrowed the scope of the evidence map. Comparisons in which HA formulation and procedural factors, such as injection schedule, volume, technique, or concomitant treatment, changed together were excluded. This trade-off was accepted to reduce procedural confounding and improve the interpretability of differences between formulations, although it does not isolate molecular weight from other product characteristics. Consequently, this review maps a defined subset of direct HA comparisons and does not comprehensively represent all clinical uses of HA in TMDs.

## 5. Conclusions

Evidence from five prospective reports comparing HA preparations under matched procedural protocols is insufficient to guide clinical decisions about HA molecular weight in TMDs. It does not establish equivalence, superiority, or comparative safety of molecular-weight categories. The principal contribution of this review is to characterize the limitations of existing comparisons, including inconsistent formulation definitions and incomplete methodological, outcome, and safety reporting, and to specify priorities for future study design and reporting.

## Figures and Tables

**Table 1:** Summary of eligibility criteria.

Category	Inclusion	Exclusion
Study design	Prospective randomized, quasi-randomized, or nonrandomized studies with a direct comparison of distinct HA preparations under the same procedural protocol, including eligible pairs within multi-arm reports.	Retrospective or noncomparative studies, case reports, and sources other than published journal articles.
Population	People of any age with clinically diagnosed temporomandibular joint (TMJ) disorders allocated to different HA products used within the same protocol.	Mixed-population studies that do not report TMJ-specific data separately; non-human studies.
Intervention	Intra-articular HA injections into the TMJ comparing two or more distinct HA products with an identical procedural protocol across the compared arms, including eligible pairwise comparisons from multi-arm studies.	Comparisons with different injection volumes, schedules, sites, techniques, or concomitant procedures; comparisons of different doses of the same preparation or combined intra-articular agents.
Comparison	Head-to-head comparisons of different HA preparations using the same injection protocol; eligible pairwise comparisons from multi-arm studies were permitted.	Comparisons of HA versus placebo/saline/other agents/procedures; any head-to-head comparison where protocols differ between HA groups.
Outcomes	At least one clinical or safety outcome: pain, mandibular function, mouth opening, global improvement, adverse events, or complications. Follow-up timing was charted.	Studies not reporting any eligible clinical or safety outcomes.
Language	No language restrictions.	

**Table 5:** Evidence gaps and proposed minimum reporting items and design priorities for future comparative studies.

Domain	Gap Identified in the Included Reports	Proposed Minimum Reporting Items and Design Priorities
HA formulation	Overlapping molecular-weight categories; missing absolute values; a discrepancy with product information; incomplete concentration and cross-linking data.	Report product name, manufacturer, absolute molecular-weight value or range (kDa), HA concentration (mg/mL), injected volume per joint per session (mL), and presence and type of cross-linking. Identify the source of these specifications and all formulation attributes that differ between groups. For cross-linked products, specify what the reported molecular-weight value describes.
Procedural protocol	Variation in injection schedules, arthrocentesis techniques, lavage volumes, and accompanying treatment across reports; incomplete procedural descriptions.	Report the injected joint compartment, number of injections per joint, injection interval, use or absence of arthrocentesis, needle technique, lavage solution and volume (mL), and concomitant treatment, including rescue medication. Hold procedural components constant between formulation groups or evaluate them as separate prespecified factors.
Study population	Chronic osteoarthritis and broader internal derangement populations; differing baseline impairment and incompletely characterized HA subgroups.	Specify diagnostic criteria, disorder subtype, eligibility criteria, symptom duration, baseline pain and functional impairment, and unilateral or bilateral involvement. Report baseline characteristics and numbers of enrolled patients and treated joints separately for each formulation.
Outcomes and analysis	Different pain contexts and follow-up times; missing subgroup denominators and dispersion estimates; bilateral joints treated as independent in one report.	Prespecify the primary outcome, measurement instrument, pain context where applicable, and primary assessment time. Define secondary outcomes and follow-up times. Report group results with dispersion, between-group effect estimates with 95% confidence intervals, and numbers analyzed for each outcome and time point by formulation. Specify analysis exclusions, missing-data handling, the unit of analysis, and how correlation between bilateral observations is addressed.
Allocation, blinding, and sample size	Predictable, sequential, or unreported allocation; incompletely described blinding; small eligible comparison groups.	Report random sequence generation, allocation concealment, who was blinded, and how blinding was implemented. Base sample size on a prespecified clinically meaningful between-group difference in the primary outcome at the primary assessment time, with stated assumptions concerning variability, statistical power, significance level, and attrition.
Safety	Systematic adverse-event ascertainment not described; formulation-specific safety results absent from two reports; one arm discontinued after reactions in 2 of 5 recipients.	Prespecify adverse-event definitions, collection methods and times, and the observation period. Report event type, onset, duration, severity, seriousness, assessed relatedness, and associated treatment discontinuation or withdrawal. For each formulation, report exposed patient and treated-joint counts, patients with at least one event, and total events. Identify the denominator for each event proportion, explicitly report zero events when applicable, and specify any safety stopping criteria.

Abbreviations: HA, hyaluronic acid; kDa, kilodaltons. The proposed items are derived from gaps identified in this review. Unavailable formulation information should be identified explicitly rather than inferred from product names or molecular-weight categories.

## Data Availability

Data are contained within the article.

## Supplementary Materials

The following supporting information can be downloaded at: https://www.mdpi.com/article/10.3390/jcm15197597/s1, File S1: Preferred Reporting Items for Systematic reviews and Meta-Analyses extension for Scoping Reviews (PRISMA-ScR) Checklist.

## Appendix A

Final database searches are provided below.

PubMed:

(“temporomandibular joint” OR TMJ OR temporomandibular) AND (hyaluronic OR “hyaluronic acid” OR HA OR hyaluronate OR “sodium hyaluronate” OR viscosupplement*) AND (injection OR intraarticular OR “intra-articular” OR arthrocentesis OR lavage) AND (compare OR comparison OR compared OR versus OR vs OR “head-to-head” OR “head to head” OR “randomized” OR “randomised” OR “trial” OR “clinical trial”) AND (“molecular weight” OR “low molecular” OR “high molecular” OR low-molecular OR high-molecular OR crosslink* OR cross-link* OR brand OR product OR formulation OR preparation OR different OR distinct)

BASE:

(“temporomandibular joint” OR TMJ OR temporomandibular) AND (hyaluronic OR “hyaluronic acid” OR HA OR hyaluronate OR “sodium hyaluronate” OR viscosupplement*) AND (injection OR intraarticular OR “intra-articular” OR arthrocentesis OR lavage) AND (compare OR comparison OR compared OR versus OR vs OR “head-to-head” OR “head to head” OR “randomized” OR “randomised” OR “trial” OR “clinical trial”) AND (“molecular weight” OR “low molecular” OR “high molecular” OR low-molecular OR high-molecular OR crosslink* OR cross-link* OR brand OR product OR formulation OR preparation OR different OR distinct)

CENTRAL (Cochrane Library):

(“temporomandibular joint” OR TMJ OR temporomandibular) AND (hyaluronic OR “hyaluronic acid” OR HA OR hyaluronate OR “sodium hyaluronate” OR viscosupplement*) AND (injection OR intraarticular OR “intra-articular” OR arthrocentesis OR lavage) AND (compare OR comparison OR compared OR versus OR vs OR “head-to-head” OR “head to head” OR “randomized” OR “randomised” OR “trial” OR “clinical trial”) AND (“molecular weight” OR “low molecular” OR “high molecular” OR low-molecular OR high-molecular OR crosslink* OR cross-link* OR brand OR product OR formulation OR preparation OR different OR distinct) in Title Abstract Keyword

Europe PMC:

(TITLE_ABS:(“temporomandibular joint” OR TMJ OR temporomandibular) OR KW:(“temporomandibular joint” OR TMJ OR temporomandibular)) AND (TITLE_ABS:(hyaluronic OR “hyaluronic acid” OR HA OR hyaluronate OR “sodium hyaluronate” OR viscosupplement*) OR KW:(hyaluronic OR “hyaluronic acid” OR HA OR hyaluronate OR “sodium hyaluronate” OR viscosupplement*)) AND (TITLE_ABS:(injection OR intraarticular OR “intra-articular” OR arthrocentesis OR lavage) OR KW:(injection OR intraarticular OR “intra-articular” OR arthrocentesis OR lavage)) AND (TITLE_ABS:(compare OR comparison OR compared OR versus OR vs OR “head-to-head” OR “head to head” OR “randomized” OR “randomised” OR “trial” OR “clinical trial”) OR KW:(compare OR comparison OR compared OR versus OR vs OR “head-to-head” OR “head to head” OR “randomized” OR “randomised” OR “trial” OR “clinical trial”)) AND (TITLE_ABS:(“molecular weight” OR “low molecular” OR “high molecular” OR low-molecular OR high-molecular OR crosslink* OR cross-link* OR brand OR product OR formulation OR preparation OR different OR distinct) OR KW:(“molecular weight” OR “low molecular” OR “high molecular” OR low-molecular OR high-molecular OR crosslink* OR cross-link* OR brand OR product OR formulation OR preparation OR different OR distinct))
